# Characterization and Structural Evaluation of Niobium-Integrated Chitosan–Gelatin Hybrid Hydrogels

**DOI:** 10.3390/gels12020107

**Published:** 2026-01-27

**Authors:** Muhammad Usman Khalid, Arunas Stirke, Martynas Talaikis, Vidas Pakstas, Tatjana Kavleiskaja, Alessandro Márcio Hakme da Silva, Wanessa De Melo

**Affiliations:** 1 Department of Functional Materials and Electronics, State Research Institute Centre for Physical Sciences and Technology (FTMC), 10257 Vilnius, Lithuania; muhammad.usmanbajwa@ftmc.lt; 2 Department of Organic Chemistry, State Research Institute Centre for Physical Sciences and Technology (FTMC), 10257 Vilnius, Lithuania; martynas.talaikis@ftmc.lt; 3 Department of Structural Analysis of Materials, State Research Institute Centre for Physical Sciences and Technology (FTMC), 10257 Vilnius, Lithuania; vidas.pakstas@ftmc.lt; 4 Institute of Chemistry, Vilnius University, 3 Saulėtekio Avenue, 10257 Vilnius, Lithuania; tatjana.krivorotova@chf.vu.lt; 5 Scientific and Technological Institute, Bioengineering Graduate Program, Brazil University, São Paulo 05508-220, Brazil; alessandro.silva@ub.edu.br

**Keywords:** niobium pentoxide, chitosan–gelatin, hydrogel, swelling, gel fraction, rheology

## Abstract

Chitosan–gelatin (CG) hybrid hydrogels are widely recognized for their biocompatibility and suitability for soft tissue engineering, wound dressings, and biomedical coatings. Despite this promise, conventional CG systems often exhibit limited mechanical strength, restricted durability, and uncontrolled swelling, which can reduce their clinical relevance. In this study, we introduce an enhanced soft hydrogel platform reinforced with niobium pentoxide (Nb_2_O_5_) nanoparticles and chemically crosslinked using glutaraldehyde, with citric acid serving as a dissolution medium and processing aid. Three hydrogel variants (G1, G2 and G3) were prepared by adjusting nanoparticle concentration and subsequently evaluated through structural, morphological, swelling, gel-fraction, and rheological analyses. SEM imaging revealed that increasing Nb_2_O_5_ content produced notable architectural transitions—from smooth porous matrices to nanoparticle-distributed, heterogenous pore structures. XRD, FTIR, and Raman spectroscopy confirmed the structural retention of Nb_2_O_5_ and its effective interaction with the polymer network. Swelling and gel-fraction measurements demonstrated improved network stability in nanoparticle-loaded systems, with G2 providing the most desirable balance between swelling capacity (298%) and gel fraction (91%). Rheological studies further identified G2 as the most stable and elastic composition, exhibiting strong shear-thinning behavior and high structural recovery. Overall, G2 emerges as the optimal formulation for future biomedical development.

## 1. Introduction

Hydrogels made from natural polymers have received considerable attention in biomedical engineering because of their biocompatibility, customizable physicochemical qualities, and structural similarities to the extracellular matrix (ECM) [1,2]. Among the numerous natural polymers used to produce hydrogels, chitosan and gelatin are among the most widely used biopolymers due to their biodegradability, bioactivity, and suitability for wound healing, drug delivery, and tissue regeneration. Chitosan, a cationic polysaccharide derived from chitin, has hemostatic, antimicrobial, and film-forming qualities, whereas gelatin, a partly hydrolyzed version of collagen, has strong cell adhesion and gel-forming capabilities. Combining chitosan and gelatin improves mechanical stability, increases elasticity, and permits the creation of multifunctional hybrid hydrogels for improved biomedical applications [3,4]. Gelatin, a denatured collagen derivative, exhibits strong cell adhesion properties and is commonly used to enhance the bioactivity of polymeric matrices. The combination of chitosan and gelatin produces a synergistic blend that resembles the natural extracellular matrix, promoting cell proliferation while retaining structural integrity [5,6].

Chitosan-based hydrogels present fabrication challenges due to the limited solubility of chitosan, which requires acidic aqueous media for dissolution. Although acetic acid is commonly employed, citric acid has gained attention as a more effective alternative. Citric acid is a naturally occurring organic acid capable of interacting with the hydroxyl and amino functional groups of biopolymers, thereby facilitating chitosan dissolution. In addition to its solubilizing role, citric acid acts as a safe and non-toxic plasticizer, improving the flexibility and handling characteristics of hydrogels, particularly for applications involving direct contact with tissues or cells [7,8,9]. Furthermore, crosslinking is a critical step in hydrogel fabrication, as it determines the mechanical strength, structural stability, and degradation behavior of the polymer network [10]. Among available crosslinking agents, glutaraldehyde remains one of the most effective and widely used for chitosan–gelatin systems, forming stable Schiff base linkages through reactions between its aldehyde groups and the amino groups of the polymers [11,12].

Incorporating inorganic nanoparticles into biopolymer hydrogels has become a widely used technique for improving their functioning. Niobium pentoxide (Nb_2_O_5_) nanoparticles are a promising yet underexplored addition. Niobium-based materials are known for their superior chemical stability, biocompatibility, and bio-inertness [13]. Recent investigations indicate that Nb_2_O_5_ has photocatalytic activity, enhanced surface hardness, and potential antibacterial effects, making it a promising material for advanced biomedical composites [14,15]. Integrating Nb_2_O_5_ nanoparticles into a chitosan–gelatin matrix, utilizing citric acid dissolution and glutaraldehyde crosslinking, represents a novel methodology that has not yet been reported.

The goal of this work is to develop a next-generation soft hydrogel by combining three functional components: citric acid for efficient polymer dissolution, glutaraldehyde for stable crosslinking, and niobium pentoxide (Nb_2_O_5_) nanoparticles as a reinforcing nanofiller. Chitosan–gelatin hydrogels were fabricated using citric acid and glutaraldehyde, with controlled Nb_2_O_5_ concentrations incorporated to tune material properties. The resulting hydrogels were then characterized through spectroscopic, morphological, swelling, gel-fraction, and rheological analyses to understand how nanoparticle loading affects structure and performance. This framework provides a basis for a tunable hydrogel system with improved stability and mechanical behavior, supporting its future potential in biomedical applications. Looking ahead, the inherent biocompatibility, tunable porosity, and shear-thinning behavior of Nb_2_O_5_-integrated CG hydrogels position them as versatile platforms for advanced biomedical innovations. For instance, their controlled swelling and high gel fraction could enable sustained drug release in targeted therapies, while the nanoparticle reinforcement may impart photocatalytic or antibacterial properties, potentially reducing infection risks in wound dressings. Future studies could explore their integration with bioactive cues, such as growth factors, to mimic dynamic ECM environments for regenerative medicine, building on recent advances in stimulus-responsive hydrogels [16].

## 2. Results and Discussion

### 2.1. SEM Analysis

**Figure 1:**
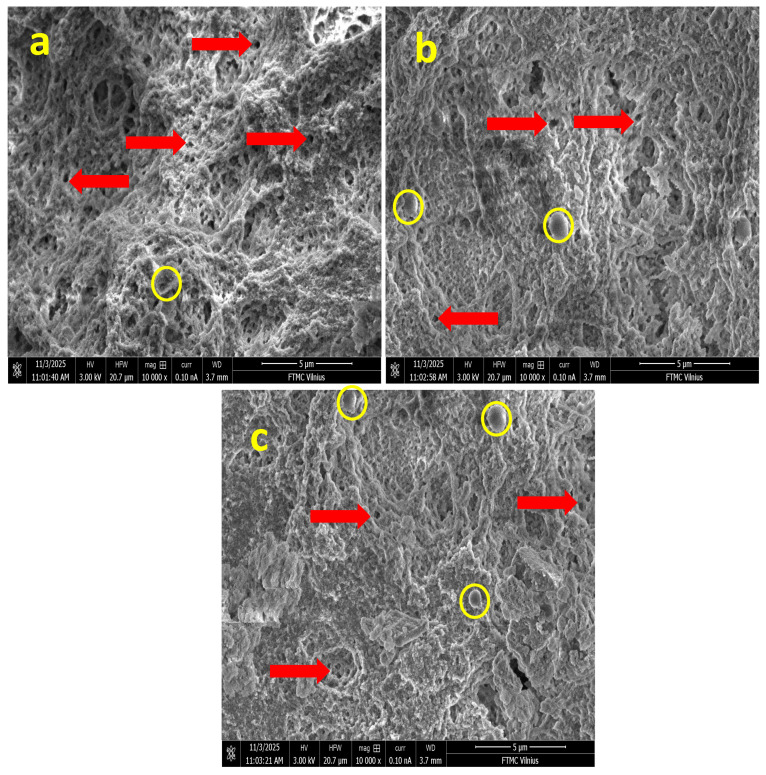
SEM images of hydrogel samples: (**a**) G1, (**b**) G2 (with arrows indicating porous gels), and (**c**) G3 (with circles highlighting spherical Nb NP agglomerates).

The internal architecture of the fabricated chitosan–gelatin (CS-Gel) hydrogels, incorporated with increasing concentrations of niobium nanoparticles (Nb NPs), was investigated using scanning electron microscopy (SEM). SEM images of hydrogel samples (a) G1, (b) G2 (with arrows indicating pores), and (c) G3 (with circles highlighting aggregated domains), as shown in Figure 1a (G1), 1b (G2), and 1c (G3), respectively, provided critical insights into the influence of inorganic nanofiller content on the morphological properties of the composite scaffolds, which are paramount for their intended biomedical applications.

All formulations exhibited a characteristic microporous, sponge-like structure, a typical outcome of the freeze-drying process, where the sublimation of ice crystals creates a three-dimensional (3D) polymeric network [17]. This observation confirms the successful formation of a porous CS-Gel matrix, a well-established scaffold base in tissue engineering that inherently supports cell adhesion and proliferation [18]. The integrity of this structure across all samples indicates that the initial polymer blending was effective.

In the sample with the lowest niobium content (G1), the matrix appeared relatively uniform, featuring well-interconnected micropores and thin, smooth polymer walls (Figure 1a). This homogeneous morphology is characteristic of a well-integrated CS-Gel blend without significant phase separation, as commonly reported in the literature [9]. The absence of pronounced inorganic domains at this low concentration suggests a fine, initial dispersion of the Nb NPs within the biopolymeric continuum, similar to the homogeneous distribution of low concentrations of silver nanoparticles in chitosan matrices observed in literature [19].

As the niobium concentration increased (G2), a distinct evolution in morphology was observed. The pore walls became noticeably rougher, and localized spherical nano-features, approximately 200–400 nm in diameter, emerged on the polymer surface as displayed in Figure 1b. These spherical domains are unequivocally attributed to agglomerates of Nb NPs embedded within the CS-Gel network. Their presence confirms the successful incorporation of the inorganic phase and suggests strong interfacial compatibility. This phenomenon mirrors findings in other composite hydrogels; for instance, reported the formation of similar nano-sized, spherical agglomerates when incorporating nano-hydroxyapatite into chitosan scaffolds, which they attributed to nanoparticle–polymer interactions and the processing method. The increased surface roughness in G2 is often desirable, as it can enhance protein adsorption and initial cell attachment, a key advantage noted for nanocomposite scaffolds [20].

The most pronounced morphological changes were evident in the highest-niobium formulation (G3). This sample displayed a more heterogeneous structure, where regions of compact, collapsed lamellar structures coexisted with highly porous zones (Figure 1c). The surface roughness was further enhanced, and a greater number of spherical Nb aggregates were visible. This heterogeneity indicates that at higher loadings, the Nb NPs significantly influence the solidification dynamics during freeze-drying. The thicker pore walls and locally densified structures are a direct consequence of stronger interfacial interactions, likely through hydrogen bonding or electrostatic forces between the Nb NPs and the functional groups (e.g., -NH_2_ of chitosan and -OH and -COOH of gelatin) [21]. These interactions can increase the local crosslinking density, effectively restrict polymer chain mobility and modify the growth pattern of ice crystals, leading to a more heterogeneous pore structure. A comparable trend was observed by [20] in chitosan–gelatin scaffolds with mesoporous silica, where higher filler content led to increased pore wall thickness and structural coarsening.

Collectively, the SEM analysis confirms that the niobium content acts as an important microstructural modulator in CS-Gel hydrogels. In G1 (low Nb_2_O_5_), the matrix is dominated by the biopolymer blend, showing minimal inorganic features. In contrast, G2 and G3 exhibit progressive integration of Nb NPs, with G2 displaying balanced nanoparticle distribution on pore walls without aggregation, while G3 shows heterogeneous densification due to higher NP clustering. This tunable morphology, achieved without collapsing the porous network, is expected to enable future tailoring of properties such as mechanical response and potential degradation rate, making these Nb-CS-Gel hydrogels a model system for advanced biomaterial development. Overall, the microstructural characteristics observed in G2 indicate a balanced integration of porosity and nanoparticle-related surface roughness, features generally associated with improved biological and antibacterial performance.

### 2.2. XRD Analysis

**Figure 2:**
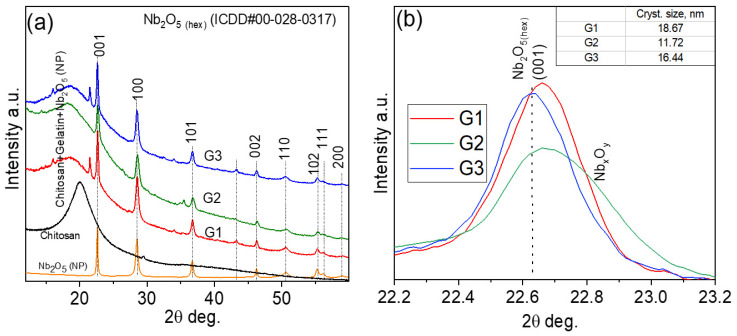
XRD patterns of Nb_2_O_5_ nanoparticles embedded in a chitosan–gelatin (**a**) XRD patterns of Nb_2_O_5_ nanoparticles, chitosan, and chitosan–gelatin mixtures containing different amounts of Nb_2_O_5_ nanoparticles (G1—5 mL, G2—7 mL, and G3—9 mL). (**b**) Fragment of the pattern showing the broadening of the hexagonal Nb_2_O_5_ (001) peak after incorporating different amounts of nanoparticles.

The structural evolution of chitosan–gelatin hydrogels integrated with varying volumes of Nb_2_O_5_ nanoparticle dispersion (G1: 5 mL, G2: 7 mL, G3: 9 mL) was probed using X-ray diffraction (XRD). Figure 2a reveals that all composites retain the characteristic reflections of hexagonal-like Nb_2_O_5_ (ICDD#00-028-0317), confirming the preservation of the crystalline phase within the biopolymer matrix. Despite the consistent phase identity, the diffraction profiles exhibit significant variations in peak intensity and breadth. The weak and broadened reflections in G1 suggest a high degree of nanoparticle dispersion or confinement by the polymer network, a phenomenon often observed at low filler loadings where the matrix dominates [22]. In contrast, the sharp and intense peaks in G3 indicate a higher degree of particle–particle interaction and micro-aggregation, a common consequence of exceeding the optimal loading capacity in biopolymer nanocomposites, as similarly documented for chitosan–TiO_2_ systems [23].

Significantly, the G2 hydrogel demonstrates an optimal structural configuration, exhibiting well-defined Nb_2_O_5_ peaks without evidence of secondary phases or significant distortion. This balanced profile indicates that the 5 mL loading fosters optimal nanoparticle distribution and polymer–particle compatibility, effectively suppressing aggregation. This finding aligns with the established concept of a critical filler concentration for achieving maximal dispersion and interfacial adhesion in hybrid networks [24].

Further insight was gained from the analysis of the (001) reflection (Figure 2b). The calculated crystallite sizes, derived from the Scherrer equation, are ~18.67 nm (G1), ~11.72 nm (G2), and ~16.44 nm (G3). The smallest crystallite size in G2 is particularly noteworthy. This suggests that the moderate nanoparticle concentration in G2 promotes effective polymer–particle interactions, where functional groups from chitosan and gelatin (e.g., -NH_2_, -OH) act as capping agents to limit crystallite growth, a mechanism previously reported for gelatin-stabilized metal nanoparticles [25]. The subsequent size increase in G3 is characteristic of coalescence and reduced matrix stabilization at high loadings [26]. In summary, the XRD analysis identifies the G2 composite (5 mL Nb_2_O_5_) as the most structurally uniform system. The optimal dispersion and minimal crystallite size achieved in this sample are expected to maximize the effective surface area of the Nb_2_O_5_ nanoparticles.

### 2.3. FTIR Analysis

**Figure 3:**
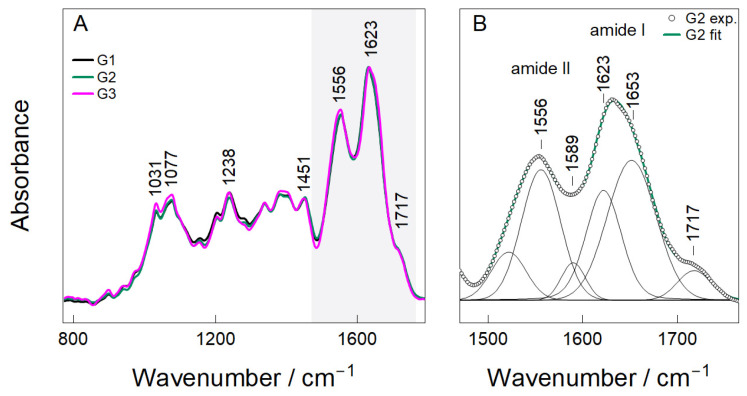
FTIR Analysis (**A**,**B**) of hydrogel samples (G1, G2, and G3).

The FTIR spectra of the hybrid chitosan–gelatin hydrogels containing different concentrations of Nb_2_O_5_ nanoparticles (G1–G3) are shown in Figure 3A,B. All samples exhibit the characteristic bands of the biopolymer network. The absorptions at 1031–1077 cm^−1^ are assigned to C–O and C–O–C stretching modes of the glucosamine backbone, whereas the peak at 1238 cm^−1^ corresponds to amide III and C–N vibrations of gelatin. The presence of these modes indicates that the polymeric matrix remains structurally intact following citric-acid dissolution and blending.

The most prominent features are located within the amide region (1500–1700 cm^−1^), where a distinct doublet centered at 1556 cm^−1^ (amide II) and 1623–1653 cm^−1^ (amide I) appears for all hydrogels (Figure 3A). Deconvolution of the G2 spectrum (Figure 3B) resolves four individual contributions at 1556, 1589, 1623 and 1653 cm^−1^, consistent with N–H bending/C–N stretching modes of amide II and C=O stretching of amide I. These assignments are in good agreement with reported spectra of chitosan-based polypeptide systems [27,28].

The component at 1623–1653 cm^−1^ also includes contributions from imine (C=N) bonds formed during the Schiff-base reaction between glutaraldehyde and primary amines. This observation confirms the formation of a chemically crosslinked chitosan–gelatin network, which aligns with the proposed synthesis mechanism. A weak additional band at 1717 cm^−1^ corresponds to residual carbonyl stretching of citric-acid-derived COOH groups, indicating that part of the citrate remains incorporated in the network as pendant functionalities that can enhance hydration and flexibility.

Although the peak positions remained essentially unchanged between G1–G3, changes in intensity and peak broadening were observed as a function of nanoparticle loading. G1 displays sharper and weaker amide bands, indicating minimal perturbation of the polymer network at the lowest Nb_2_O_5_ concentration. In contrast, G2 shows the most pronounced and symmetric amide envelope, suggesting a favorable balance between glutaraldehyde crosslinking and secondary interactions between Nb_2_O_5_ and functional groups in the matrix. At the highest concentration (G3), amide bands become broader and less well defined, consistent with increased heterogeneity and the possible onset of nanoparticle aggregation. In addition, citric acid serves primarily as a dissolution aid and plasticizer for chitosan, with FTIR indicating residual -COOH groups as non-covalent contributors to hydration rather than primary crosslinking [29]. It does not form covalent links but enhances flexibility via ionic interactions. Overall, these results indicate that Nb_2_O_5_ acts primarily through coordination and hydrogen bonding rather than forming new covalent linkages within the hydrogel architecture.

### 2.4. RAMAN Analysis

**Figure 4:**
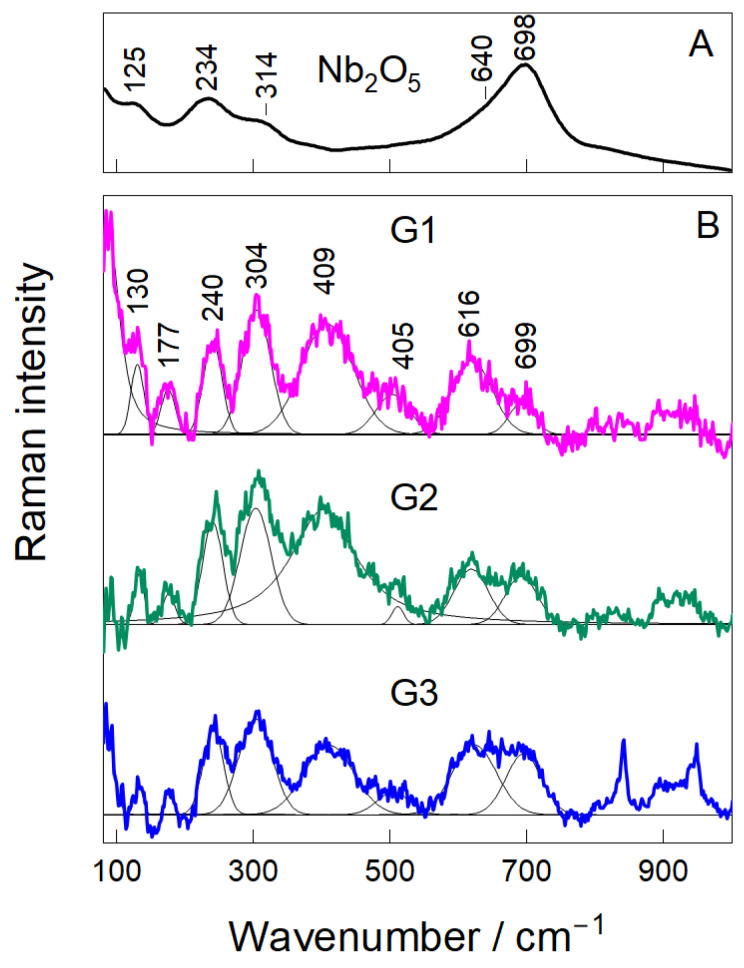
Raman Analysis (**A**,**B**) of hydrogel samples (G1, G2, and G3).

Raman spectroscopy was utilized to explore the structural integrity of the Nb_2_O_5_ nanoparticles and their dispersion state within the chitosan–gelatin hydrogel. The reference spectrum of pure Nb_2_O_5_ (Figure 4) displayed characteristic vibrational modes at 125, 234, and 314 cm^−1^, corresponding to lattice and Nb–O–Nb bending modes [30,31,32]. Two prominent peaks at 640 and 698 cm^−1^ were also observed, originating from the symmetric stretching of terminal Nb=O and bridging Nb–O–Nb bonds, respectively, which are typical fingerprints of crystalline Nb_2_O_5_ [33]. The spectra of the composite hydrogels (G1–G3) preserved all characteristic Nb_2_O_5_ bands, confirming that the nanoparticles retained their crystalline structure after incorporation into the polymeric matrix [30,32]. For the G1 sample, pronounced peaks were observed at 130, 177, 240, 304, 405, 616, and 699 cm^−1^. These bands are associated with the bending and stretching vibrations of distorted NbO_6_ octahedra, a common feature in nanostructured Nb_2_O_5_ [31,34]. The presence of these well-defined peaks indicates that even at the lowest concentration, the niobium oxide phase remains stable and is well-dispersed within the network.

The band shape and intensity provided insights into the dispersion quality and interfacial interactions. At the intermediate loading (G2), the Nb_2_O_5_ peaks became broader and more symmetric, with a relative increase in intensity compared to the polymer background. This suggests a more uniform distribution of nanoparticles and stronger interfacial interactions with the amino and carbonyl groups of the chitosan–gelatin matrix [33,35], a finding consistent with the optimal balance suggested by the FTIR analysis. For the G3 sample, the Nb_2_O_5_ peaks remained present but became notably broader and less defined, particularly in the 600–750 cm^−1^ region. This loss of spectral resolution is indicative of nanoparticle aggregation at the highest concentration.

### 2.5. Swelling and Gel Fraction Analysis

The swelling behavior and structural integrity of hydrogels are critical determinants of their potential in biomedical applications, as they directly influence solute transport, mechanical stability, and the release of bioactive agents [12]. The equilibrium swelling ratio (SR) and gel fraction (GF) of the chitosan–gelatin–Nb_2_O_5_ hydrogels (G1, G2, G3) were evaluated in phosphate-buffered saline (PBS, pH 7.4) at 37 °C to simulate physiological conditions. A control hydrogel without nanoparticles (G0) was synthesized and tested to establish a baseline and accurately quantify the impact of Nb_2_O_5_ incorporation.

The results, summarized in Table 1, reveal a clear trend influenced by the nanoparticle content. The G0 hydrogel exhibited the highest equilibrium swelling ratio of 412 ± 15%. This high swelling capacity is characteristic of a hydrophilic polymer network with a moderate crosslink density. Upon the incorporation of Nb_2_O_5_ nanoparticles, a significant and systematic decrease in the swelling ratio was observed. The G1 hydrogel (lowest Nb_2_O_5_ content) showed a swelling ratio of 385 ± 12%, while the G2 hydrogel demonstrated a further reduced SR of 298 ± 9%. The G3 sample, with the highest nanoparticle loading, displayed the most restricted swelling, showing a value of 235 ± 14%. This inverse relationship between nanoparticle content and swelling capacity can be attributed to two primary factors. First, the Nb_2_O_5_ nanoparticles act as physical crosslinking points within the polymeric matrix [36].

The surface of Nb_2_O_5_ can interact with functional groups of the biopolymers (e.g., -NH_2_ of chitosan and -C=O of gelatin) via hydrogen bonding and electrostatic interactions, effectively creating additional junctions that restrict chain mobility and reduce the free volume available for water penetration. Second, a higher effective crosslinking density, as corroborated by the gel fraction results, inherently leads to a tighter network with reduced swelling potential [12].

The gel fraction, which represents the insoluble, crosslinked portion of the hydrogel, provides direct insight into the network’s structural stability. As shown in Table 1, the gel fraction increased from 78 ± 2% for the control hydrogel (G0) to a maximum of 91 ± 2% for the G2 formulation. This significant enhancement confirms that the incorporation of Nb_2_O_5_ nanoparticles reinforces the hydrogel network. The nanoparticles not only provide physical crosslinks themselves but may also promote a more efficient and homogeneous glutaraldehyde-mediated crosslinking process by creating a favorable spatial organization of the polymer chains [37]. The optimal balance achieved in G2 results in a network with high integrity and minimal soluble, un-crosslinked polymer content.

However, at the highest nanoparticle loading (G3), the gel fraction decreased slightly to 88 ± 4%. This non-monotonic trend is a classic indicator of nanoparticle aggregation at high concentrations [38]. As suggested by the Raman analysis, Nb_2_O_5_ aggregates in G3 act as physical defects within the matrix, which can locally disrupt the continuity of the polymer network and create pathways for polymer chain extraction during the drying process, thereby lowering the measured gel fraction.

**Figure 5:**
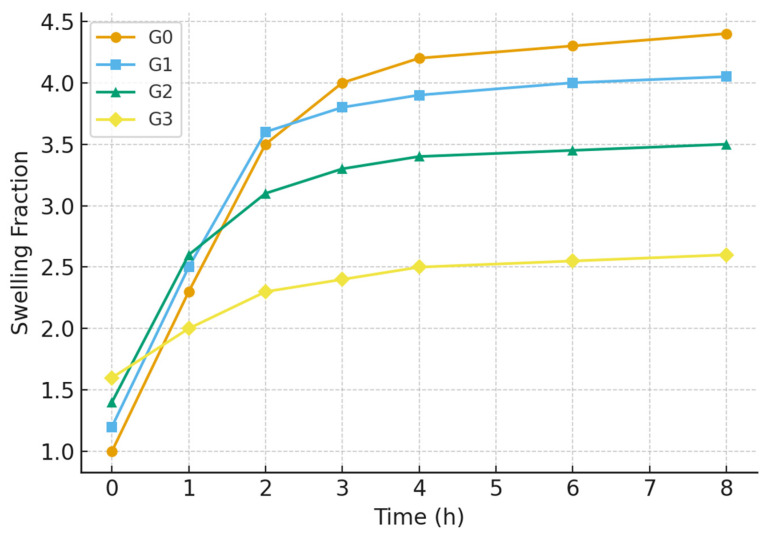
Swelling kinetics of the hydrogels (G0, G1, G2, G3).

The swelling kinetics, illustrated in Figure 5, further support these findings. All hydrogels exhibited a rapid initial swelling phase, followed by a gradual approach to equilibrium. The G2 hydrogel not only reached a lower equilibrium swelling but also did so with a more controlled kinetic profile compared to G0 and G1, which swelled rapidly and could be prone to mechanical weakening. The G3 hydrogel showed the slowest swelling rate, consistent with its highly restricted network morphology due to excessive nanoparticle filling.

In summary, the swelling and gel fraction studies demonstrate that Nb_2_O_5_ nanoparticles play a crucial role in modulating the hydrogel’s aqueous and structural properties. The G2 formulation, with an optimal nanoparticle concentration, achieves an ideal balance: a sufficiently high gel fraction (91%) ensuring structural robustness, coupled with a moderate swelling ratio (298%) that is conducive to applications requiring controlled fluid uptake. The compromised performance of G3 underscores the detrimental effects of nanoparticle aggregation, reaffirming that G2 represents the most compositionally balanced and effective hydrogel composite.

### 2.6. Rheological Analysis

#### 2.6.1. Amplitude Sweep Analysis

Rheological measurements were carried out to evaluate the mechanical integrity of the Nb_2_O_5_-reinforced chitosan–gelatin hydrogels. The amplitude sweep test monitored the evolution of the storage modulus (G′) and loss modulus (G″) as a function of strain to determine the linear viscoelastic region (LVR) and the yield point. For all formulations, G′ exceeded G″ within the LVR, confirming a predominantly elastic response and the formation of stable three-dimensional crosslinked networks [39].

**Figure 6:**
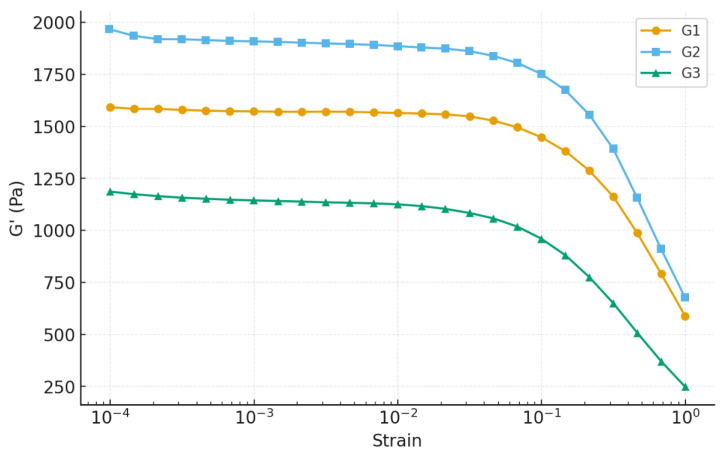
Amplitude sweep analysis of hydrogels.

A clear dependence on Nb_2_O_5_ loading was observed, with the results summarized in Figure 6. Gel G2 demonstrated the highest mechanical rigidity, exhibiting the greatest plateau modulus (G′ ≈ 2250 Pa) and preserving its LVR up to γ ≈ 0.02. This indicates a highly interconnected and resilient network capable of accommodating deformation without structural collapse. In contrast, G1 displayed a lower G′ plateau (~1430 Pa), suggesting a less dense polymeric network. Gel G3 exhibited the lowest plateau modulus (~1260 Pa) and an early decrease in G′, indicative of reduced crosslink density and earlier onset of structural degradation under stress [40].

The loss factor (tan δ) further supports these observations: G2 presented the lowest values (0.06–0.07), demonstrating dominant solid-like behavior [41], whereas G1 showed intermediate values and G3 approached the elastic–viscous transition (tan δ ≈ 0.09). This trend indicates that nanoparticles not only reinforce the G2 matrix but also impart superior mechanical stability compared to both the lower and higher nanoparticle concentrations [42,43].

The superior performance of G2 can be attributed to optimal nanoparticle–polymer interactions. Nb_2_O_5_ nanoparticles can introduce additional physical and hydrogen-bond crosslinking sites within the biopolymer chains, increasing the network connectivity and resistance against structural breakdown. However, excessive nanoparticle loading in G3 likely promotes aggregation, producing localized stress concentrations and microphase inhomogeneities that reduce mechanical resilience, a well-documented phenomenon for nanocomposite hydrogels [44,45]. Overall, the amplitude sweep analysis identifies G2 as the mechanically superior formulation, providing the best balance between stiffness, elasticity, and resistance to deformation. This rheological behavior is particularly desirable for biomedical hydrogels, which must withstand handling, mechanical loading, and cyclic deformation during application [45].

#### 2.6.2. Frequency Sweep Analysis

**Figure 7:**
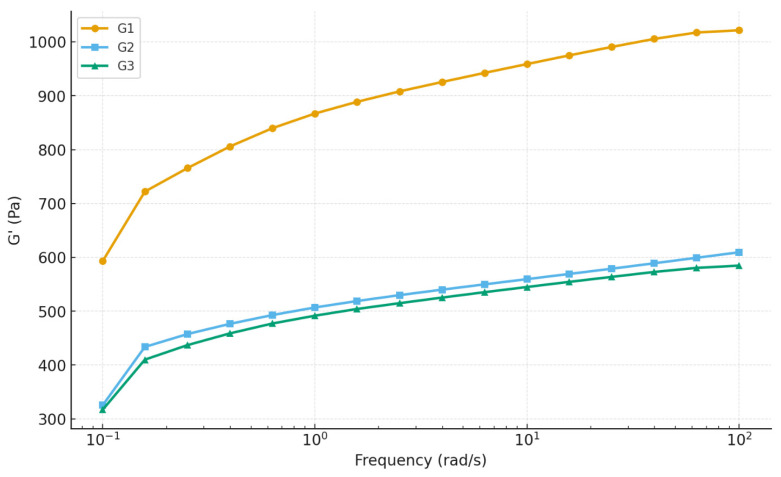
Frequency sweep analysis of hydrogels.

The viscoelastic behavior of the three hydrogel formulations (G1, G2, and G3) was characterized by oscillatory frequency-sweep tests over 0.1–100 rad·s^−1^. All gels displayed solid-like behavior throughout the frequency range: the storage modulus (G′) remained consistently higher than the loss modulus (G″), which confirms the formation of stable gel networks as shown in Figure 7. This finding aligns with typical observations for physically crosslinked polymer hydrogels and nanocomposite systems, where G′ > G″ over broad frequency ranges is taken as the hallmark of gel-like behavior [46]. Despite the common gel-like character, the magnitude and frequency response of G′ varied substantially among the three formulations. At 10 rad·s^−1^, G1 exhibited the highest G′ (~959 Pa), followed by G2 (~614 Pa) and G3 (~549 Pa). All gels hardened with increasing frequency, characteristic of polymer networks with transient cross-links. However, the degree of stiffening was most pronounced for G2 (~84% increase), less for G3 (~82%), and lowest for G1 (~72%). This suggests that G2’s network structure is more dynamic and capable of reinforcing under deformation, possibly due to favorable polymer–nanoparticle interactions and optimal nanoparticle distribution that enhance network adaptability under load. The loss factor (tan δ) remained below 0.4 for all gels, denoting strong elastic dominance. Moreover, tan δ values converged at mid-to-high frequencies (in the range relevant for application), indicating comparable elastic behavior across the gels under typical operational conditions. In other words, regardless of their absolute stiffness, all gels would feel similarly solid-like under rapid deformations.

A second frequency sweep was performed to evaluate the structural stability of the hydrogels under repeated deformation. G2 showed the strongest recovery and structural enhancement, with G′ values shifting upward during the second sweep. This demonstrates that G2 can reorganize and reinforce its internal network after being subjected to shear. In comparison, G1 exhibited a slight reduction in G′, indicating that the internal structure is more prone to irreversible weakening. G3 showed only minimal change, suggesting a stable but less adaptable structure [47].

**Figure 8:**
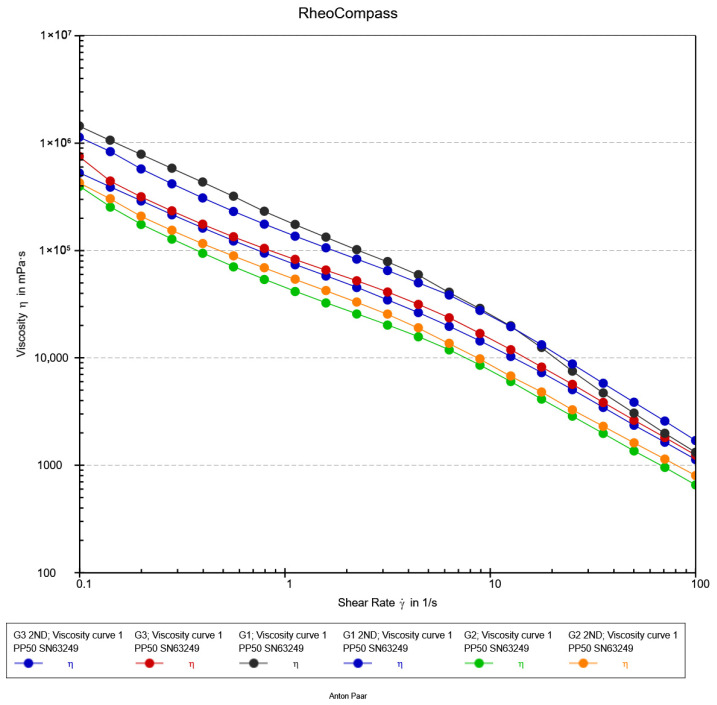
Flow Curve analysis of hydrogels.

Overall, the frequency-sweep results confirm that G2 offers the most balanced mechanical performance. Although G1 exhibits the highest initial stiffness, its limited recoverability reduces its functional reliability. G2 combines sufficient stiffness with the greatest ability to retain and even improve its structure following deformation. Therefore, G2 can be identified as the most robust and durable hydrogel formulation under dynamic loading conditions. The storage modulus variation with frequency is shown in Figure 8. These findings are in line with broader observations from previous studies of polymer and nanocomposite hydrogels, confirming that nanofiller-mediated reinforcement can significantly enhance mechanical performance but only when optimized for concentration and network homogeneity.

#### 2.6.3. Flow Curve Analysis

The flow behavior of the hydrogel formulations (G1, G2, and G3) was characterized using steady shear rheology to assess their processability and injectability, a critical attribute for modern minimally invasive procedures. As illustrated in Figure 8, all formulations demonstrated a pronounced shear-thinning response, with the apparent viscosity decreasing by nearly three orders of magnitude over the investigated shear rate range (0.1–100 s^−1^). This non-Newtonian behavior is characteristic of self-assembled and physically crosslinked networks, where applied shear forces induce a reversible breakdown of the microstructure, aligning polymer chains and disrupting non-covalent interactions to facilitate flow [48].

The viscosity profiles exhibited a consistent and pronounced hierarchy, with values decreasing in the order G3 > G1 > G2 across the full shear range. This trend underscores how subtle variations in nanocomposite formulation, such as nanoparticle concentration and dispersion, can precisely tune the density and relaxation dynamics of the hydrogel network [49]. The robust viscosity of G3 indicates a more restricted network, whereas the fluidity of G2 under shear suggests an optimally percolated structure that yields efficiently. The practical implications of these flow curves are paramount. The high structural viscosity at low shear rates (<1 s^−1^) ensures shape retention and stability post-injection, a key requirement for maintaining a defined scaffold at the target site [50]. Crucially, the behavior in the high-shear regime (10–100 s^−1^) directly correlates with injectability. The superior shear-thinning of G2 predicts a significantly lower extrusion force, essential for preserving cell viability in injectable biomimetic materials [51,52,53]. This is essential not only for clinician handling but also for preserving the viability of sensitive encapsulated therapeutic agents, such as cells or proteins, by minimizing shear-induced damage during the injection process [49].

Overall, the steady-shear analysis confirms that all formulations are potent shear-thinning hydrogels. The distinct hierarchy in their flow profiles highlights the critical role of compositional optimization. Formulation G2, with its optimal balance of low high-shear viscosity and inherent structural integrity, emerges as the most promising candidate for advanced injectable biomaterial applications.

## 3. Conclusions

In this study, a novel series of chitosan–gelatin (CG) hydrogels reinforced with niobium pentoxide (Nb_2_O_5_) nanoparticles were successfully fabricated and comprehensively characterized. The incorporation of Nb_2_O_5_ proved to be an effective strategy for enhancing the structural and functional performance of the CG network. Structural, morphological, and spectroscopic analyses confirmed the successful integration and uniform distribution of the nanoparticles within the polymer matrix, resulting in strong interfacial interactions that underpin the improved material properties. Nanoparticle concentration was identified as a key determinant of the final hydrogel characteristics. While all Nb_2_O_5_-reinforced formulations outperformed the nanoparticle-free CG hydrogel, the intermediate concentration in the G2 formulation provided the most balanced network. This was reflected in its higher storage modulus, improved yield behavior, and favorable shear-thinning profile, together with an optimal combination of swelling capacity and structural integrity as evidenced by the gel-fraction results.

Overall, the G2 hydrogel successfully addresses the common limitations of conventional CG systems and demonstrates a combination of mechanical stability, controlled swelling, and injectable behavior. These Nb_2_O_5_-reinforced CG hydrogels not only address mechanical limitations of traditional systems but also open avenues for multifunctional applications. Speculatively, the nanoparticle-mediated enhancements in elasticity and structural recovery could support load-bearing roles in soft tissue scaffolds, while their injectable nature may facilitate minimally invasive delivery for cartilage repair or dermal fillers. Grounded in their bio-inert Nb_2_O_5_ component and porous architecture, these materials hold promise for personalized medicine, such as 3D-printed implants with tailored degradation profiles. Ongoing work will validate these prospects through biological and in vivo assessments.

## 4. Materials and Methods

Chitosan (low molecular weight, 70% deacetylated), gelatin from porcine skin, citric acid anhydrous, and glutaraldehyde solution (25% in H_2_O) were purchased from Sigma-Aldrich Merck KGaA, Darmstadt, Germany. Niobium pentoxide (Nb_2_O_5_) nanoparticles (<100 nm particle size) were obtained by following the synthesis method as explained [14]. All other chemicals were of analytical grade. Deionized (DI) water was used throughout the experiments.

### 4.1. Synthesis of Chitosan–Gelatin–Niobium (CG-Nb) Hybrid Hydrogels

**Figure 9:**
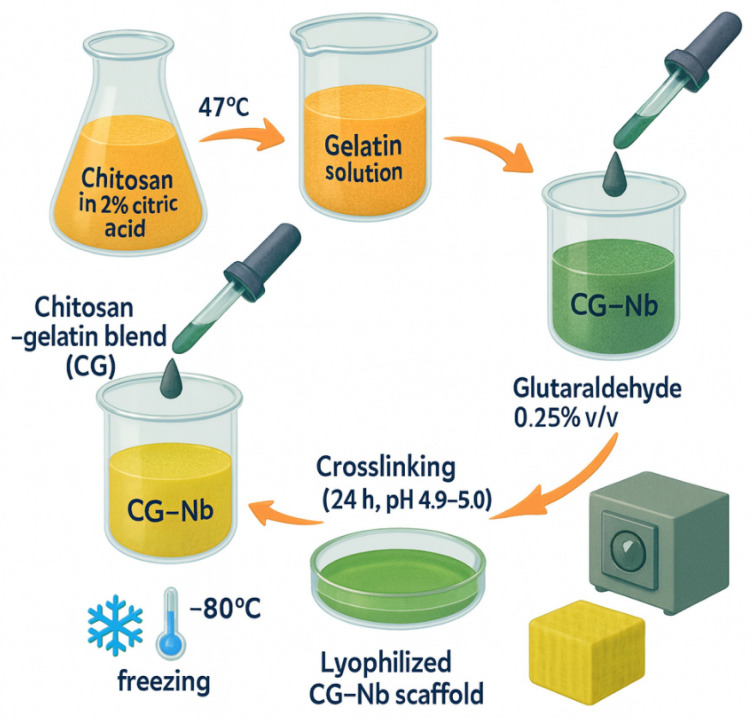
Synthesis overview steps used to produce the CG–Nb soft hybrid hydrogel.

**Figure 10:**
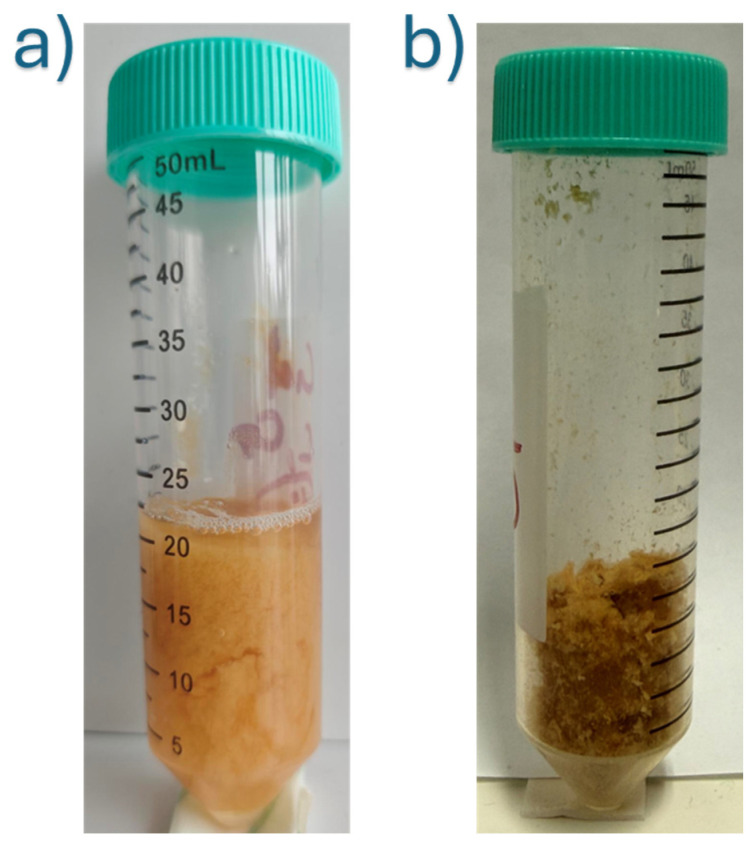
CG-Nb hydrogels: (**a**) Hydrated forms, (**b**) Freeze-dried scaffold.

The hybrid hydrogels (CG-Nb) were synthesized via a sequential method involving dissolution, blending, crosslinking, and nanoparticle incorporation, as shown in Figure 9. A 1% (*w*/*v*) chitosan solution was prepared by dissolving 500 mg of chitosan in 50 mL of a 2% (*w*/*v*) aqueous citric acid solution. The mixture was stirred continuously at 47 °C for 18–24 h to achieve complete dissolution, leveraging citric acid as an effective solvent and plasticizer [7] separate 8% (*w*/*v*) gelatin solutions were prepared by dissolving 400 mg of gelatin in 5 mL of DI water at 50 °C. The warm gelatin solution was then added dropwise to the chitosan–citric acid solution, which was stirred vigorously, resulting in a homogeneous chitosan–gelatin (CG) blend. A stable suspension of Nb_2_O_5_ nanoparticles (2 mg/mL) was prepared in DI water using probe sonication (200 W, 10 min, ice bath). Predetermined volumes of the 2 mg/mL Nb_2_O_5_ suspension were added to separate batches of the CG blend to achieve three distinct nanocomposite formulations: 5 mL (10 mg Nb_2_O_5_) for G1, 7 mL (14 mg Nb_2_O_5_) for G2, and 9 mL (18 mg Nb_2_O_5_) for G3. Given that each hydrogel batch contained a total of 900 mg of biopolymer (500 mg chitosan + 400 mg gelatin), the final Nb_2_O_5_/(chitosan + gelatin) weight ratios were approximately 1.1% *w*/*w* (G1), 1.6% *w*/*w* (G2), and 2.0% *w*/*w* (G3). Subsequently, glutaraldehyde was added to each mixture at a concentration of 0.25% (*v*/*v*) of the total solution volume to act as the primary crosslinking agent [10]. The reaction mixture was stirred for another 1 h to ensure homogeneity. The pH of the final mixtures was adjusted to 4.9–5.0 to optimize the Schiff base reaction between glutaraldehyde and the polymer amines. The solutions were then transferred to Petri dishes and allowed to crosslink for 24 h at room temperature, forming stable hydrogels (G1, G2, G3). The resulting gels were thoroughly washed with DI water to remove any unreacted glutaraldehyde. For subsequent characterization, the hydrated hydrogels were frozen at −80 °C for 6 h and then lyophilized for 48 h to obtain dry, porous scaffolds. The hydrated hydrogels displayed a soft, translucent appearance, which converted into porous scaffolds following freeze-drying (Figure 10).

### 4.2. Crosslinker Selection (GA)

The use of glutaraldehyde (GA) as a crosslinker in this study requires specific consideration. GA is a highly efficient crosslinking agent that provides stable covalent networks, ideal for fundamental structure–property investigations as conducted here [54]. However, its known cytotoxicity poses a significant limitation for direct clinical translation. In this proof-of-concept study, GA was selected for its reliability in forming a robust network to isolate the reinforcing effect of Nb_2_O_5_ nanoparticles. For any future development aimed at direct biomedical application, replacing GA with more biocompatible alternatives (e.g., genipin, EDC/NHS, or physical crosslinking methods) would be an essential subsequent step. The rigorous washing protocol employed aimed to remove residual GA, though quantitative assessment of residues is recommended in future biocompatibility studies.

### 4.3. Characterization

Scanning Electron Microscopy (SEM): The surface morphology of the hydrogels was evaluated using a Helios Nano Lab 650 SEM (FEI, Hillsboro, OR, USA), equipped with a Schottky field-emission electron source and operated at an accelerating voltage of 3 kV. The system includes a gallium FIB column and an Oxford Instruments X-Max EDS detector for elemental analysis. The instrument provides a resolution of 0.8–1.5 nm across 30 kV to 200 V operation ranges. All images were acquired under high-resolution mode using a working distance of 3.7 mm to ensure optimal surface detail while minimizing charging.

X-ray Diffraction (XRD): XRD patterns of the studied powders were measured using an X-ray diffractometer Smart Lab (Bruker AXS GmbH, Karlsruhe, Germany) equipped with an X-ray tube with a 9-kW rotating Cu anode. The measurements were performed using Bragg–Brentano geometry with a graphite monochromator on the diffracted beam and a step scan mode with a step size of 0.02° (in 2θ scale) and a counting time of 1 s per step. The measurements were conducted in the 2θ range of 10–75°. Phase identification was performed using the software package PDXL 2.9 (Rigaku) and the ICDD powder diffraction database PDF4+ (2024 release). The size of the Nb_2_O_5_ crystallites was calculated based on the broadening of XRD peaks using the graphical Halder–Wagner method implemented into PDXL software.

Fourier Transform Infrared (FTIR) and Raman Spectroscopy: Infrared absorption spectra were collected using an Alpha FTIR spectrometer (Bruker, Germany) equipped with a diamond attenuated total reflectance (ATR) accessory and a room temperature detector DLATGS. The spectral resolution was set to 4 cm^−1^, and 50 scans were co-added for sample and background channels. Raman measurements were performed using the Raman microscope in Via (Renishaw, Gloucester, UK), equipped with a thermoelectrically cooled (−70 °C) CCD camera and an 830 nm laser line (10 mW at the sample) and an 830 lines/mm grating. The objective lens (20×/NA 0.4, Leica) focused laser light onto a line of approximately 80 × 15 μm in size. The total acquisition time of a single spectrum was 30 min. Experimental infrared and Raman spectra were deconvoluted using Gaussian-Lorentzian shape components.

Swelling and Gel Fraction Studies: The equilibrium swelling ratio was determined in PBS (pH 7.4) at 37 °C. Pre-weighed dry samples (Wd) were immersed for 24 h, then re-weighed (Ws) after blotting. The swelling ratio was calculated as [(Ws − Wd)/Wd] × 100. The gel fraction was determined by weighing the dried samples after extraction in DI water at 50 °C for 24 h (We) relative to the initial weight (Wi): (We/Wi) × 100.

Rheological characterization: Rheological measurements were carried out using a rotational rheometer (Anton Paar PP50) equipped with a parallel-plate geometry (50 mm diameter and 1.0 mm gap) at 37 °C to simulate physiological conditions. The hydrogels (G1, G2, G3) were placed on the lower plate and allowed to equilibrate before measurement. All measurements were conducted in triplicate (*n* = 3), and data are presented as mean ± standard deviation. First, an amplitude sweep (0.01–100% strain) was performed at a fixed angular frequency of 1 rad·s^−1^ to determine the linear viscoelastic region (LVR) and the critical strain for network breakdown. Next, a frequency sweep in the range of 0.1–100 rad·s^−1^ was carried out within the LVR to determine the storage modulus (G′), loss modulus (G″), and complex viscosity. Finally, steady-shear flow curves were obtained by measuring the apparent viscosity as a function of shear rate (0.1–100 s^−1^) to evaluate the pseudoplastic behavior and flowability of the hydrogels.

## Figures and Tables

**Table 1:** Swelling and gel fraction properties of the hydrogels.

Sample	Nb_2_O_5_ Loading	Swelling Ratio (%)	Gel Fraction (%)
G0	0%	412 ± 15	78 ± 2
G1	~1.1%	385 ± 12	82 ± 3
G2	~1.6%	298 ± 9	91 ± 2
G3	~2.0%	235 ± 14	88 ± 4

## Data Availability

All data generated or analyzed during this study are included in this published article.
